# Adaptation of the Clamp and Button Sutures to Prolapse of the Vagina

**Published:** 1858-01

**Authors:** Horatio R. Storer

**Affiliations:** Boston


### ART. III.—*Adaptation of the Clamp and Button Sutures to Prolapse of the Vagina.* By HORATIO R. STORER, M.D., of Boston.

CASE I.—Mrs. W——, a servant of Dr. Morland, was placed under my charge by that gentleman in the summer of 1856.

June 3d. Patient a widow, aged forty-two, general health tolerably good, though suffers somewhat from dyspepsia. Has for a long time noticed pain in right shoulder and left iliac region, which last has always been more or less tender on pressure. Ceased menstruating, she is confident, thirteen years since, at which time, if her age as given is correct, she could have been but twenty-nine. Has seen no colored discharge since. Has had, till of late, but little leucorrhœa. Has had but one child, a son, some twenty years ago; easy labor, but rapid, especially toward its close. Has never miscarried.

Thinks her bowels are freely moved daily. Urine passed with great difficulty, but little in the twenty-four hours, though constant sense of distended bladder. Has noticed this symptom gradually increasing, till for past three months has found it unbearable. A great sense of fullness and distention in vagina, which at its mouth is blocked by a projecting mass. This by its size and sensitiveness seriously impedes walking, and at other times often causes severe pain, particularly when rising up and sitting down. Some pain in back, and sensation of dragging in loins. Is confident she has prolapsus uteri. Has never allowed an examination by a physician.

Chest auscultated and percussed; its organs healthy.

Upon examination, abdomen of ordinary size and fullness; no tenderness on pressure except over left ovary, as she had stated. Vagina found completely filled by a large and fluctuating tumor, at once increased by coughing or forcing down, or by rising to the feet; behind this the finger passed readily, uterus being perfectly in place. Vaginal gape large, perineum almost entirely effaced. Rectum, supposed by her empty, was found by the finger in vagina crammed with hardened fæces so as closely to simulate carcinoma, till firm pressure produced their characteristic œdematous feel. The free action of an enema, which was attended by vomiting and syncope, made the diagnosis more certain, and a catheter easily emptied the prolapsed and distended bladder. A little fleshy protuberance, size of a bean, behind meatus and on urethra, but external to it; this would have seemed like an urethral pouch from dilatation, could the catheter have been made to enter it. The patient entered the Lying-in Hospital on June 8th, and was then seen by my colleagues, Drs. Read and Dupee. To the fleshy tubercle above described, which she had often inspected and been greatly troubled by in mind, I applied a ligature, on June 17th, with the assistance of Dr. Read; it was once subsequently tightened, and came away on the seventh day. From the time of her entrance a doubly-curved catheter with a flexible tube attached was retained in the bladder, and plugs of lint soaked in a saturated solution of tannin, as advised by Dr. Fordyce Barker of New York, for prolapsed uterus, were kept in the vagina night and day. She was put on simple tonics with house diet, and rapidly improved in spirits, the displacement daily diminishing in extent. She was at all times disposed to despond, frequently had her sleep broken by incubus, and on June 12th she got a slight attack of diarrhœa, but this was easily checked.

On the 25th of June she was discharged much improved.

Re-entered the hospital on July 26th.

Has continued tonics till present time. General health better. Has found much relief from the absence of the mass removed by ligature, but complains that the fullness at vulva, dysuria, and difficulty in walking have returned. Upon examination bladder found again down. Will not allow pessary, whether of India-rubber, pig's bladder, or aught else. Is satisfied that all other means are but palliative, and begs if any operation can give chance of relief that it may be performed.

The procedure recommended and practiced with such success by Mr. Brown of London was, therefore, decided on, with certain modifications suggested to me a short time previously by my friend Dr. Marion Sims of New York.

July 27th. Low diet. Pill of oxgall, grs. x., to-night and to-morrow night.

July 29th. Bowels cleared by an enema in forenoon. Proceeded to operate at 4 P.M. with the assistance of Dr. Read, the patient having been etherized. Strips of mucous membrane, an inch in length by half an inch in breadth, were first pared away on each side just within and parallel to the nymphæ, their edges being brought together by two linen stitches. Then beginning just posteriorly to the lower margin of these patches, the membrane was freely dissected up and removed, in shape of a horse-shoe, and entirely around the posterior fourchette to the depth of nearly an inch within vagina. This having been done, ligatures of silver wire, three in number, were introduced, secured upon perforated German-silver quills, and clamped tightly by shot.

Very little blood was lost, and the patient slept quietly through the operation.

Sims' catheter having been introduced and patient put upon her side, sol. morphiæ, gtt. xx., were administered, morphiæ sulphat., gr. $\frac{1}{2}$, introduced as suppository, and a wet compress applied to the wound.

10 P.M. Comfortable.

July 30th. Slept but little last night; disturbed by restless infant in next room, and besides made effort to keep awake lest she should disturb ligatures. No pain; pulse not raised; no tenderness of abdomen. Thighs to be kept together by napkin round knees and ankles, unless patient complains. Ice to be freely given, opiates and suppository as required, and dressing continued to the wound. Thin gruel sparingly.

July 31st. Comfortable night. Complains at times of flatulence; this relieved by opiate and carminative. Urine profuse; skin cool; very little heat about pudenda; pulse quiet. In evening slight nausea.

August 1st. Complains greatly of flatulence. Abdomen hardly enlarged. Violent retching at times, with expulsive efforts to empty rectum; these not controlled by suppository. Repeat carminative, apply sinapism, and subsequently poppy fomentations to abdomen.

At 3 P.M., distress increasing, passed hollow bougie into rectum, with escape of much fetid gas, and instant relief.

At 7 P.M., complaining of general soreness of limbs; removed to softer mattress, and at 10 P.M. felt perfectly at ease.

August 2d. Slept quietly till 3.30 A.M., when had profuse watery and horribly offensive fæcal discharge, attended with escape of but little free flatus, and no pain. Complains of still further desire, so passed bougie and drew off much more fluid of similar character. Ordered chalk mixture with laudanum, catechu and charcoal, to be given as required. Wound healthy. Pulse quiet; as yet has never exceeded 84. No tenderness of abdomen; some faintness and hiccough.

At night dejections continuing, ordered brandy ʒi. every half-hour; and, alternating with a pill of two grains of tannin and one of opium, ʒi. of powdered charcoal in boiled milk; one of the two every hour and a half.

August 3d. Had ten dejections yesterday and five last night, offensive and containing blood. Very low throughout day, and allowed to see clergyman. Pulse, which had flagged for last twenty-four hours, begins to come up toward night. Now learn that four hours before operation, and contrary to strict orders that diet should be low, simple, and preparatory, which orders the matron had endeavored rigidly to enforce, patient got access to and devoured several mouthfuls of *lobster*, for which she had always insatiable longing.

August 4th. Diarrhœa still continues; five dejections yesterday and two last night, much the same in character, but beginning to show the charcoal and to lose their fœtor; still much blood, and at intervals flatus. Now allowed to pass water herself, but in recumbent posture. Slept most of night. At 8 A.M. transient flush from stimulants; pulse 120. At 10 A.M. pulse 110, skin quite cool, respiration hurried, much hiccough and sighing; gets but little sleep. Discontinue charcoal and substitute wine whey and wine with arrowroot, for brandy. Pills as before, every three hours. Slight but not unhealthy looking purulent discharge from wound; no heat; no fœtor.

August 5th. Patient feebler; diarrhœa continuing, five dejections during night. Was put last evening on sulphate of copper, half a grain every two hours, without apparent effect; therefore substitute for each dose three grains of subacetate of lead.

Has had for past forty-eight hours after every dejection the ordinary starch and laudanum enema, instead of which now gets one containing a scruple and a half of tannin; this given every half hour if not retained.

The matron of the hospital had also now been suffering for two days with dysenteric symptoms, bloody stools, tenesmus, &c., the effect of close attendance upon this patient; treated by Dr. Read.

9 P.M. From twelve to fifteen dejections during day, many of them involuntary, their odor less fetid; some tenesmus. Injections last ordered not retained; their strength to be doubled. Some little tendency to delirium this evening, and subsultus tendinum. Pulse for last thirty-six hours about 120. Great despondency.

August 6th. Weaker. Discharges involuntary, seven last night. Injections not retained; add to each, cupri. sulph. grs. iss. All medicine by mouth to be discontinued, and beef-tea freely given.

Enema retained from 9.30 to 1.30; after this discharges returning as before. Beef-tea greatly relished, though tongue quite dry. Resume last pills at night.

Aug. 8th. Yesterday and to-day much as before; discharges frequent, and with much blood and mucus. Pulse 106.

Aug. 9th. No discharge from 11.30 A.M. to 4.30 P.M. Disposed to sleep, and stupid; opium diminished by one-half.

Aug. 10th. Two discharges last night, more fæcal in character. Weather the past week very adverse; wet, cold, and lowering.

Aug. 11th. Pulse down to 100; brighter. Sutures removed to-day. Small and sharply marked slough under right quill, wound itself seeming to have nearly healed; dressed with diluted liq. sodæ chlorinat. In passing suppository yesterday, rectum carefully examined and found unaffected. No dejection from 11 yesterday morning till 10 to-night.

Aug. 12th. Two dejections during night, in color, consistence, and odor more fæcal; none to-day. Evidently failing, though rallies at times. Discontinue all medicine and give stimulants with care.

Aug. 13th. Very low at 7 A.M. Rallied at 9, and then nearly as for past three days. Has had no rigor since operation. At 4.30 P.M. the sphincters were relaxed, urine and fæces were profusely passed, and at 6 she died.

A woman having just been confined in next room, and there being no dead-house attached to the hospital, an autopsy was not made.

The patient was seen during her sickness by Drs. Read, Dupee, and Storer, Sen., in consultation; by Dr. Morland and by Dr. Oliver.

CASE II.—Mrs. Mary C., aged thirty-five, was delivered on Dec. 5th, 1856, by a midwife, who used much violence toward the close of the labor, the pains being very slight. Child a boy, of moderate size. The patient had previously been confined but once, ten years previously, of a girl, and had never miscarried.

I saw her for the first time on Dec. 23d, eighteen days subsequent to her confinement. At that time a laceration of the perineum was found, extending completely to the sphincter ani, and a little beyond it on one side; its edges much swollen, sore, and excessively sensitive to the touch. Suppuration profuse. Much flatus passed during the examination. Urine under control. Profuse diarrhœa. Nursing, though but little milk.

On Feb. 4th, 1857, a careful examination was again made. No restoration of the perineum had taken place, the edges of the wound were much enlarged, protruding like hemorrhoids, and the patient was complaining of great bearing down and discomfort.

Objection being made by her to the necessary operation, a perineal bandage was advised and worn for the ensuing four months, with but partial relief. By this time the protrusion of the vaginal walls had so increased, both laterally and posteriorly, that the patient returned to me at the Eustis Street Dispensary, to request a radical cure. This was accordingly attempted on June 8th, with the assistance of Dr. Hayward of Roxbury.

Operation at 3 P.M., patient having been prepared by a several days' course of ox-gall. She was etherized, put on her back as for lithotomy, the dissections made as in the former case, save that those anteriorly were omitted as here unnecessary, and the silver wires were passed. Instead, however, of being fastened to metal quills, their ends were passed through a leaden shield, as recommended by Bozeman, of Alabama, for vesico-vaginal fistula, and were tightly clamped upon it by shot. This shield or "button" was an inch and a half in length by a half-inch in breadth, and was oval in form. The treatment immediately subsequent to the operation was as in the former case, with the addition of confining the legs in a more immoveable position, by attaching the heels to a belt around the waist. Water dressings were alone applied.

No unpleasant symptom intervened, and on the 14th, six days after operation, the button was removed. Union firm and complete. No suppuration from beside wires. On the next and following day, the wires were removed. On the 16th, a profuse and painful dejection, without at all disturbing the integrity of the adhesions.

June 17th. Felt so well that she has been up to-day, contrary to orders, and has used active exertion. Last night loosed the guards from her knees, and waking up in fright suddenly spread her thighs apart, causing, however, but a slight notch at the anterior edge of cicatrix.

June 22d. Parts firm and tough; union to full depth of wound; perineum of normal extent. Patient finds bearing down gone, and expresses herself to be as comfortable when on feet as before accident.

For some time subsequently, micturition continued frequent and painful, especially after over-fatigue; but this yielded in great measure to simple treatment. Oct. 10th, four months after operation, on examination for the ordinary irritable tumor of the meatus, the perineum was found still perfect. The local discomfort was gone; the patient was cured.

Case I., though fatal, furnished rather ground in favor of than against the operation performed. It was matter of no little regret to me at the time that *post-mortem* dissection seemed unjustifiable, especially as I had been strongly dissuaded from operating by a friend who had just refused the attempt to a patient of his own; but so far as could be judged by the condition of the wound, from the outset to the very end, the absence of local symptoms, the extent to which adhesion seemed to have taken place, the apparently insufficient cause from this source for the dysentery, the apparently more than sufficient cause for it from another, there seems reason to believe that had the patient obeyed orders, she would not only have lived, but been cured.

The case is published on the ground so seldom heeded, that where successful cases have been reported, the surgeon is bound to withhold no failure, especially when attended by death.

Case II., on the other hand, was successful from the outset; and this, too, though under apparently less favorable circumstances than the other. Of a naturally much more irritable, impatient, and restless temperament, nursing throughout her confinement to bed, over-anxious about the child and the success of the operation, this patient seemed much more likely to go wrong.

In neither case was that course taken—entire division of the sphincter ani—so strongly insisted on by Mr. Brown as all important to success. I believe that with the old quill suture it is an unnecessary, unscientific, and cruel complication, and that with the modifications of suture here adopted it becomes unjustifiable. With Mr. Brown's other suggestions as to the nature and general treatment of both cystocele and rectocele, typical cases of each of which have been here presented, one cannot too fully concur.

The above cases, so far as they go, may serve to show the adaptability of the two new sutures, now so celebrated in connection with vesico-vaginal fistula, to the operation for lacerated perineum. The old quill suture, however used, whether with ligatures of hemp or silk, or the less irritating ones of silver or lead, secured by twisting, had always this disadvantage, that however closely the deeper portions of the wound were brought into contact, its upper edges would necessarily gape to a greater or less degree; an evil requiring and yet not fully overcome by the interrupted suture between the quills. The substitution in practice of Sims' clamp proved, as I had expected, to be attended with certain advantages; but these have been far excelled by the button of Bozeman, which for neatness, simplicity, completeness of action, and ease of application, I have found everything that could be desired.

